# Gas Chromatography–Mass Spectrometry-Based Metabolomic Analysis of Wagyu and Holstein Beef

**DOI:** 10.3390/metabo10030095

**Published:** 2020-03-06

**Authors:** Tomoya Yamada, Mituru Kamiya, Mikito Higuchi

**Affiliations:** Division of Livestock Feeding and Management, National Agriculture and Food Research Organization, Nasushiobara, Tochigi 329-2793, Japan; m_kamiya@affrc.go.jp (M.K.); mikito@affrc.go.jp (M.H.)

**Keywords:** metabolome, beef, Wagyu, Holstein

## Abstract

Japanese Black cattle (Wagyu) beef is characterized by high intramuscular fat content and has a characteristic sweet taste. However, the chemical components for characterizing the sweet taste of Wagyu beef have been unclear. In this experiment, we conducted a metabolomic analysis of the longissimus muscle (sirloin) in Wagyu and Holstein cattle to determine the key components associated with beef taste using gas chromatography–mass spectrometry (GC-MS). Holstein sirloin beef was characterized by the abundance of components such as glutamine, ribose-5-phosphate, uric acid, inosine monophosphate, 5-oxoproline, and glycine. In contrast, Wagyu sirloin beef was characterized by the abundance of sugar components (maltose and xylitol). Dietary fat is known to increase the intensity of sweet taste. These results suggest that the sweet taste of Wagyu beef is due to the synergetic effects of higher sugar components and intramuscular fat.

## 1. Introduction

Food quality, especially the taste of food, is affected by numerous chemical components. Metabolomic analysis has been used to select biomarkers from numerous metabolites. Therefore, the relationship between metabolomic profiling and food quality has been investigated to identify quality-related components. Previous reports showed that the metabolomic profiles of foods such as fermented alcoholic beverages [1], soybeans [2], and tomatoes [3], strongly affect their taste.

Metabolomic analyses of meat have also been conducted to identify quality-related components. The relationships between metabolomic profiles and processing conditions of hams [4], the muscle type of pork [5], and the sensory perceptions of pork [6] have been reported. In addition, metabolome studies of beef have shown that metabolomic profiles were affected by geographical origin [7], breed [8,9], storage conditions [10], and aging periods [11].

Japanese Black cattle, also called Wagyu, are characterized by their great capacity for intramuscular adipose tissue accumulation [12,13]. The high intramuscular adipose tissue content of beef, called marbling, improves the texture, juiciness, and tenderness of Wagyu beef [13]. In sensory tests, Wagyu beef had significantly higher sensory characteristic scores than beef from other cattle breeds [14,15]. Interestingly, Wagyu beef has a characteristic sweet aroma and sweet taste that are not detected in other cattle breeds in sensory tests [15,16,17]. Previous reports indicated that the sweet aroma of Wagyu beef was affected by the lactone and decenal components [18,19]. However, the metabolomic biomarkers discerning the breed differences in beef, especially the characteristic sweet taste of Wagyu beef, have remained unclear. Holstein cattle are categorized as a dairy breed, and Holstein beef is characterized as lean meat [20,21]. Previous sensory test results have indicated breed differences between sensory characteristic scores of Wagyu and Holstein beef [14,15]. Therefore, to elucidate the breed differences in beef taste, a comparison of Wagyu and Holstein is thought to be the optimal model. In the present study, we conducted a metabolomic analysis of longissimus muscle (sirloin) samples from Wagyu and Holstein cattle to identify the metabolomic biomarkers characterizing the breed differences in beef taste.

## 2. Results

**Figure 1:**
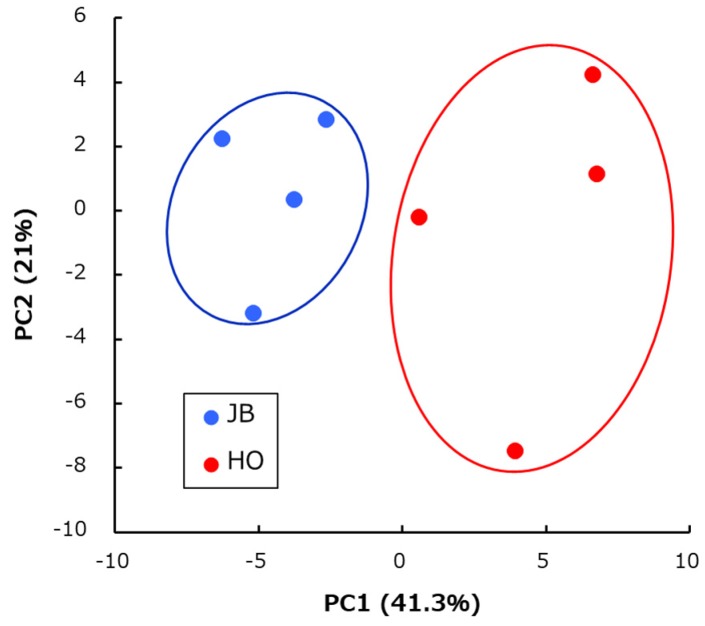
Principal component analysis (PCA) of metabolome data from Japanese Black Wagyu (JB) and Holstein (HO) sirloin samples. ●: JB (*n* = 4), ●: HO (*n* = 4).

**Figure 2:**
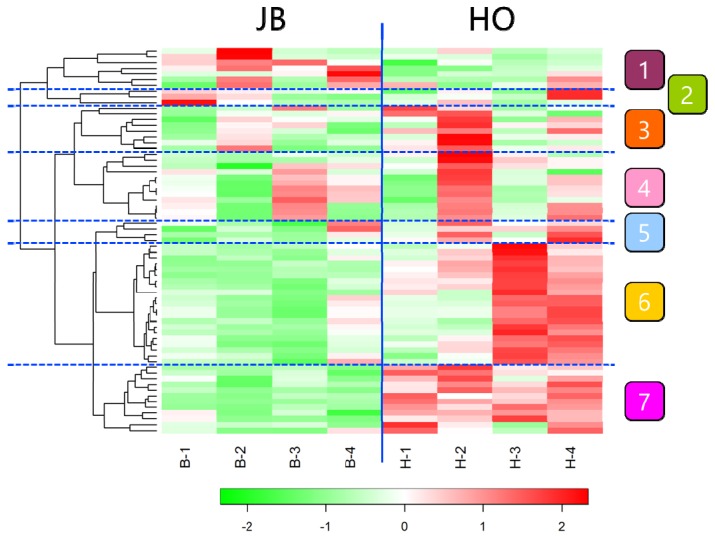
Heatmap of metabolites in Japanese Black Wagyu (JB) and Holstein (HO) sirloin samples. The upregulated metabolites are shown in red, and the downregulated metabolites are presented in green. Cluster 1, which characterized the Wagyu sample, was mainly composed of maltose, xylitol, stearic acid, palmitic acid, and nonanoic acid. Cluster 7, which characterized the Holstein sample, was mainly composed of proline, glycine, succinic acid, amino propanoic acid, creatinine, pyruvic acid, inosine, and ribose. B1-B4: Japanese Black Wagyu (*n* = 4); H1-H4: Holstein (*n* = 4).

Gas chromatography–mass spectrometry (GC-MS) analysis detected 67 metabolites in the sirloin samples of Wagyu and Holstein. Full results are shown in Supplementary Table S1. The principal component analysis (PCA) score plots showed that the metabolomic profile was divided into Wagyu and Holstein groups (Figure 1). The heatmap of metabolites also showed a difference between Wagyu and Holstein groups (Figure 2). Metabolites contributing to cluster 7, which characterized the Holstein sample, were mainly composed of amino acids (proline and glycine), amino compounds (succinic acid, amino propanoic acid, creatinine, and pyruvic acid), and nucleic acid metabolites (inosine and ribose). In contrast, cluster 1, which characterized the Wagyu sample, was mainly composed of sugar components (maltose and xylitol) and fatty acids (stearic acid, palmitic acid and nonanoic acid). Table 1 shows the differences in the relative quantity of the main metabolite compounds in Wagyu and Holstein samples. The amount of glutamine, ribose-5-phosphate, uric acid, inosine monophosphate, 5-oxoproline, and glycine in Holstein samples was significantly higher than in Wagyu samples. In contrast, the amount of maltose and xylitol in Wagyu samples was significantly higher than that in Holstein samples.

## 3. Discussion

In the present study, we showed that the Holstein sirloin samples were characterized by amino acids, amino compounds and nucleic-acid metabolites. We also showed that the relative amount of glutamine, ribose-5-phosphate, uric acid, inosine monophosphate, 5-oxoproline, and glycine in the Holstein sirloin samples was significantly higher than in the Wagyu samples. The abundance of amino acids, amino compounds, and nucleic-acid metabolites clearly reflects the lean meat content of Holstein beef. Previous studies also showed the relationship between metabolomic profiling and beef quality [7,8,9,10,11]. These results indicate that metabolomic analysis is an optimum approach to identify quality-related chemical components of beef. The cooking of meat forms a characteristic taste via numerous chemical reactions. The Maillard reaction is one of the major chemical reactions of cooked meat that produces many flavoring components [22]. Amino acids and nucleic-acid metabolites are main meat-flavor precursors for the Maillard reaction [23]. Therefore, a higher amount of amino acid and nucleic-acid metabolites in Holstein beef indicates the abundance of a Maillard reaction substrate. These results suggest that breed differences in beef metabolomic profiles affect the taste of cooked meats. The elucidation of metabolomic profile differences between Wagyu and Holstein cooked meat is an important subject for further study.

We showed that the Wagyu sirloin samples were characterized by sugar components and fatty acids. The present study also showed that the relative amount of maltose and xylitol in Wagyu sirloin samples was significantly higher than that in Holstein samples. Ueda et al. reported that the relative amount of malic acid, maltose, trehalose, arabitol, isomaltose, n-acetylserine, and inositol in Wagyu beef was significantly higher than that in Holstein beef [9]. The aging periods of beef affect the meat quality and metabolomic profile [11]. The difference between the results of our study and those of Ueda et al. would be attributed to meat aging conditions. The meat aging period in the present study was at 4 °C for 7 days after slaughter. In contrast, the aging in Ueda et al. was at 4 °C for 20 days [9]. On the other hand, results showing a higher amount of maltose in Wagyu beef than in Holstein beef were common to both experiments. These results suggest that a higher maltose concentration is a primary feature of Wagyu beef, independent of the aging period. The causative substance of the sweet taste of Wagyu beef remains unclear. Amino acids have different taste properties depending upon their chemical structure [24]. Glycine is categorized as a “sweet” amino acid [24]. The present results showed that glycine was abundant in the Holstein sirloin samples. Therefore, the possibility that amino acids contribute to the sweet taste of Wagyu beef would be excluded. In contrast, the present study showed that maltose and xylitol, categorized as sugar components, are abundant in Wagyu beef. Wagyu is characterized by higher intramuscular fat content than Holstein [12,13]. Previous reports indicated that dietary fat increased the intensity of sweet taste [25,26]. These results suggest that the sweet taste of Wagyu beef is affected by the synergetic interaction between higher sugar components and intramuscular fat. Threshold sweetness concentrations of maltose and xylitol have been reported using sensory test methods [27,28]. Kearsley et al. reported that the threshold sweetness concentration of maltose was 1.07% (w/v), and that of xylitol was 0.51% (w/v) [27]. In molar volumes, Birch et al. showed that the threshold sweetness concentration of maltose was 21.0 mM/l, and that of xylitol was also 21.0 mM/l [28]. However, the effect of dietary fat on the threshold sweetness concentration of sugar components has not been reported. The slaughter age of Wagyu (aged 29–30 months) and Holsteins (aged 21–22 months) in this study was defined in accordance with the commonly applied fattening periods of each breed in Japan. Ueda et al. also analyzed beef samples of Wagyu (aged 31–32 months) and Holsteins (aged 21 months) that were similar to the age of cattle used in this study [9]. Previous studies have shown that the slaughter age affects meat quality and sensory traits of beef [29,30]. To our knowledge, there are no previous studies examining the effects of slaughter age on metabolomic profiling of beef. Therefore, the differences between fattening periods of Wagyu and Holsteins in this study may affect the metabolomic profiling of beef. In addition, there is a possibility that other metabolites, which we could not detect in this study, might affect the sweetness of beef. Further studies are needed to clarify the effects of metabolites on the sweet taste of Wagyu beef.

## 4. Materials and Methods

### 4.1. Animals

Wagyu steers (aged 29–30 months, *n* = 4) and Holstein steers (aged 21–22 months, *n* = 4) were used in this study. They received a concentrate (78% total digestible nutrients and 13% crude protein) and orchard grass hay (56% total digestible nutrients and 8% crude protein) ad libitum from 10 months of age until they were slaughtered. Longissimus muscle (sirloin) samples were collected at slaughter. The sirloin samples (1.5 kg) were collected between the third and fourth lumbar vertebrae from the left side of the carcass. Samples were vacuum-packed and wet-aged at 4 °C for 7 days and then stored at −80 °C for later metabolome analysis. All animals received humane care as outlined in the Guide for the Care and Use of Experimental Animals (No.1631B004, National Agriculture and Food Research Organization).

### 4.2. GC-MS Analysis

Frozen sirloin samples were powdered with liquid nitrogen and weighed (100 mg) in the frozen state. Frozen samples were plunged into 80% methanol and homogenized using zirconia beads and an ultrasonic homogenizer for 5 min. The samples were centrifuged at 15,000 rpm for 5 min. The supernatant was filtered using a Mono-Spin C18 column (GL Science, Tokyo, Japan), and then the filtration (50 µL) was dried by a nitrogen gas flow. Methoxyamine hydrochloride solubilized with pyridine (20 mg/mL, 50 µL) was added to each sample, and oxime formation was achieved by reacting at 30 °C for 90 min. Trimethylsilyl-trifluoroacetamide (50 µL) was then added to each sample, and trimethylsilylation was carried out by reacting at 37 °C for 30 min. Analyses were performed on a gas chromatography–mass spectrometer (GC-MS, QP2010Ultra, Shimadzu, Kyoto, Japan) using a DB-5 column (Agilent Technologies, Santa Clara, CA, USA) at the Kazusa DNA Research Institute. The carrier gas was helium in a flow of 1.1 mL/min. The injection temperature was 280 °C, and the injection volume was 0.5 µL. The temperature program was isothermal for 4 min at 100 °C, then raised at a rate of 4 °C / min to 320 °C and held for 8 min. The ion source temperature and scan speed were set to 200 °C and 2500 u/sec, respectively. Sample peaks were recorded over the mass range of 45–600 *m/z*. The retention time correction of peaks was carried out based on the retention time of a standard alkane series mixture (C-7 to C-33). Annotation and relative quantification of metabolite was measured by each peak using the GC-MS solution (Shimadzu) and GC/MS Metabolite Database Ver. 2 (Shimadzu). The relative area was calculated using the peak area of each metabolite relative to the analyzed sample weight at Kazusa DNA Research Institute.

### 4.3. Statistical Analysis

Principal component analysis (PCA) was conducted using SampleStat software (Human Metabolome Technologies, Japan). Hierarchical cluster analysis (HCA) was performed and heatmap formation analyzed using PeakStat software (Human Metabolome Technologies). Differences between the relative quantity of metabolites in Wagyu and Holstein samples were evaluated using Welch’s t-test. Results are presented as means, and S.D. values of *p* < 0.05 were considered significant.

## Figures and Tables

**Table 1:** Main metabolite compounds in Japanese Black Wagyu and Holstein sirloin samples.

Change		Relative Area				Comparative Analysis	
	Compound Name	JB		Ho		JB/Ho	
		Mean	S.D.	Mean	S.D.	Ratio	*p*-Value
Increase	Maltose	7.1 × 10^4^	1.4 × 10^4^	4.0 × 10^4^	1.5 × 10^4^	1.8	0.022 *
	Xylitol	2.7 × 10^4^	4.4 × 10^3^	1.8 × 10^4^	2.3 × 10^3^	1.5	0.022 *
	Palmitic acid	8.8 × 10^4^	3.6 × 10^4^	6.0 × 10^4^	8.8 × 10^3^	1.5	0.221
	Stearic acid	6.7 × 10^4^	4.0 × 10^4^	5.2 × 10^4^	1.3 × 10^4^	1.3	0.513
	Ribose	4.3 × 10^5^	1.2 × 10^5^	3.5 × 10^5^	5.6 × 10^4^	1.2	0.264
	Sedoheptulose7-phosphate	7.9 × 10^4^	3.2 × 10^4^	6.5 × 10^4^	2.3 × 10^4^	1.2	0.513
	Mannose	1.1 × 10^6^	2.7 × 10^5^	8.9 × 10^5^	2.8 × 10^5^	1.2	0.406
	Glycerol 3-phosphate	2.4 × 10^4^	7.9 × 10^3^	2.1 × 10^4^	3.3 × 10^3^	1.1	0.623
Decrease	Glycine	1.6 × 10^6^	1.1 × 10^5^	2.6 × 10^6^	2.4 × 10^5^	0.6	0.001 **
	Ornithine	3.9 × 10^4^	6.3 × 10^3^	6.7 × 10^4^	2.3 × 10^4^	0.6	0.094
	5-Oxoproline	9.2 × 10^4^	9.2 × 10^3^	1.7 × 10^5^	3.2 × 10^4^	0.6	0.014 *
	Inosine monophosphate	1.8 × 10^5^	7.9 × 10^4^	3.3 × 10^5^	1.4 × 10^4^	0.5	0.030 *
	Uric acid	1.1 × 10^4^	1.4 × 10^3^	2.2 × 10^4^	4.8 × 10^3^	0.5	0.014 *
	2-Hydroxyglutaric acid	5.8 × 10^3^	2.0 × 10^3^	1.3 × 10^4^	5.3 × 10^3^	0.5	0.075
	Ribose 5-phosphate	3.2 × 10^4^	5.4 × 10^3^	6.9 × 10^4^	1.2 × 10^4^	0.5	0.004 **
	Glutamine	3.5 × 10^4^	3.8 × 10^3^	9.8 × 10^4^	3.1 × 10^4^	0.4	0.026 *

Values are expressed as means and S.D. ratio: fold intensity of metabolite compounds (JB/Ho). Annotation and relative quantification of metabolites was measured by each peak using the gas chromatography–mass spectrometry (GC-MS) solution (Shimadzu) and GC/MS Metabolite Database Ver. 2 (Shimadzu). Japanese Black Wagyu (JB, *n* = 4), Holstein (HO, *n* = 4) * *p* < 0.05, ** *p* < 0.01.

## Supplementary Materials

The following are available online at https://www.mdpi.com/2218-1989/10/3/95/s1, Table S1: Metabolites of Wagyu and Holstein Beef.
