# Supplementary material for: Gas Chromatography–Mass Spectrometry-Based Metabolomic Analysis of Wagyu and Holstein Beef
Source: Metabolites. 2020 Mar 6;10(3):95. doi: 10.3390/metabo10030095 (PMC7142765; doi:10.3390/metabo10030095)
Supplement: Supplementary file 1 [file metabolites-10-00095-s001.pdf]

Supplementary Table 1. Metabolites of Wagyu and Holstein Beef.

Metabolite	Wagyu-1	Wagyu-2	Wagyu-3	Wagyu-4	Holstein-1	Holstein-2	Holstein-3	Holstein-4	Average (Wagyu)	Average (Holstein)	StdDev (Wagyu)	StdDev (Holstein)
Boric acid-3TMS	108491	129988	124724	135018	166437	172015	125108	99914	124555.1235	140868.4345	11504.60382	34397.27463
Pyruvic acid-meto-TMS	7087	6350	7397	5209	11188	10712	8743	8293	6510.790228	9733.906998	972.5009425	1429.795727
Alanine-2TMS	5758760	5581943	4857522	6202538	7068333	7472708	9466398	8209049	5600191.002	8054122.014	559718.9804	1053304.093
3-Hydroxypropionic acid-2TMS	9845	9014	6620	6281	7657	8217	7363	12018	7940.009177	8814.00901	1758.375672	2165.400405
3-Hydroxybutyric acid-2TMS	29073	39894	42822	27856	33321	56655	40045	42525	34911.25791	43136.70267	7555.991768	9815.683717
Valine-2TMS	131562	100640	96764	127391	132084	146569	173327	181684	114089.4794	158416.1312	17918.89585	23075.81775
Urea-2TMS	1087770	1021686	914316	1003546	652169	1016388	740401	1446654	1006829.353	963903.0824	71511.29179	357274.1087
4-Hydroxybutyric acid-2TMS	68953	82758	75189	84952	89792	101276	88398	119253	77962.78716	99679.36094	7319.135333	14267.64098
Leucine-2TMS	1233053	1092716	966212	1413790	1261932	1250119	1815195	1810407	1176442.676	1534413.248	192133.2932	321496.5438
Glycerol-3TMS	985978	1296178	1223429	1181738	1213475	1526082	1060654	1337328	1171830.663	1284384.893	132619.8134	196896.2014
Phosphoric acid-3TMS	5513416	5396048	5484001	5727029	8736379	8028158	7568643	8318202	5530123.568	8162845.308	140421.5227	491356.4529
allo-Isoleucine-2TMS	642740	530836	475911	674584	636051	641474	834455	914212	581017.6821	756548.0312	93327.35052	139867.8866
Isoleucine-2TMS	642740	530836	475911	674584	636051	641474	834455	914212	581017.6821	756548.0312	93327.35052	139867.8866
Proline-2TMS	502822	391883	397310	378730	513982	541535	667016	558215	417686.1981	570187.1137	57290.76578	67079.73464
Succinic acid-2TMS	177895	127854	125999	212561	282671	197191	158027	283541	161077.1908	230357.5583	41903.52539	62973.17839
Glycine-3TMS	1580013	1503791	1764038	1649784	2645712	2263360	2836678	2621037	1624406.576	2591696.632	110544.0835	239164.9071
Glyceric acid-3TMS	24545	28112	42572	24743	46966	35189	25057	31778	29993.15792	34747.29931	8544.063696	9168.891637
Nonanoic acid-TMS	14350	44802	19640	38887	38011	17896	27526	38046	29419.67284	30369.64036	14708.255	9678.023853
Serine-3TMS	534221	503060	455372	659837	606621	599154	1133447	906471	538122.4325	811423.1579	87381.97888	258027.9885
Threonine-3TMS	74301	70254	64028	87330	101437	100026	141010	122303	73978.53894	116194.2008	9853.071868	19427.84673
3-Aminopropanoic acid-3TMS	247282	236421	243450	204654	370371	272552	210065	322381	232952.001	293842.0747	19393.7961	68660.86838
Niacinamide-TMS	104516	98280	115214	110954	179156	132581	144109	177880	107240.7922	158431.7926	7418.102566	23672.43475
Malic acid-3TMS	102056	185748	87179	95131	145032	196181	127510	143065	117528.486	152947.1623	45884.09931	29869.36869
Methionine-2TMS	16416	17355	15051	21946	18584	15940	27610	28891	17691.91886	22756.20936	2989.807672	6456.741418
5-Oxoproline-2TMS	82415	86904	99632	100792	135925	155448	211179	169470	92435.72956	168005.6756	9176.638194	31900.5277
4-Aminobutyric acid-3TMS	24929	37175	31466	21796	41640	47291	28955	48814	28841.51477	41674.9454	6862.202659	9024.142109
Cysteine-3TMS	74929	72657	55302	99940	74996	82082	116190	104496	75706.96017	94441.00073	18380.41272	19191.96153
Creatinine-3TMS	3057912	1991838	2872729	2244925	3604715	4427858	2873285	3780283	2541850.962	3671534.903	505463.0612	639144.8583
2-Hydroxyglutaric acid-3TMS	7517	7517	3529	4643	11368	6640	19518	13388	5801.378021	12728.58135	2032.403716	5337.039605
Hypotaurine-3TMS	22773	20863	21030	24850	23419	41771	29794	27047	22379.01287	30507.91937	1860.355966	7949.739115
Ornithine-3TMS	41209	36758	31692	46365	46108	63327	99837	57341	39006.07005	66653.08761	6259.549563	23245.71342
Glutamic acid-3TMS	5303	4779	5164	6240	5122	6023	10213	7064	5371.556514	7105.658575	619.6632182	2218.427903
Phenylalanine-2TMS	298201	269099	256083	349479	311468	295712	405103	412437	293215.6703	356179.8093	41436.20039	61138.74443
Asparagine-3TMS	44428	38019	32553	44443	40592	57117	79481	71868	39860.65645	62264.65695	5734.313322	17173.62281
Taurine-3TMS	528392	755071	217664	493440	654737	1538811	279627	423283	498641.9214	724114.4944	220315.3687	564686.6598
Ribose-meto-4TMS	360227	357621	400437	608835	280658	349486	340231	417247	431780.0627	346905.1686	119652.6795	55939.89078
Xylitol-5TMS	23217	29620	22552	31193	16256	16117	19361	20695	26645.49692	18107.17132	4398.566875	2284.704195
Glutamine-4TMS	32646	30961	39478	36053	66519	95599	140036	88000	34784.37167	97538.54871	3778.550293	30892.16953
Putrescine-4TMS	29207	29502	28404	36508	33465	45393	31228	46414	30905.53501	39124.93993	3763.934688	7891.005129
Glycerol 3-phosphate-4TMS	35104	22524	18725	18244	21071	25759	17829	20881	23649.12738	21385.04967	7872.811299	3272.645496
Hypoxanthine-2TMS	431018	522360	456897	687969	529320	597579	596117	655573	524561.1084	594646.9923	115521.7107	51608.70822
Arginine-3TMS	18643	22530	18590	28231	23759	30274	42687	32717	21998.46181	32359.11557	4546.134929	7854.812351
Ornithine-4TMS	70297	54041	80826	69745	93784	191141	94812	58515	68727.24233	109563.099	11038.95943	56942.81863
Fructose-meto-5TMS(1)	661048	439588	831962	745895	499429	881554	550658	706765	669623.2537	659601.6707	168484.6626	172251.2272
Fructose-meto-5TMS(2)	442314	307814	566889	511890	345108	585310	371109	485239	457226.8245	446691.3134	111893.3736	110653.8352
Mannose-meto-5TMS(1)	1039327	728081	1373858	1109241	597751	1266681	774374	917746	1062626.697	889138.0026	265519.4663	283686.9156
Glucose-meto-5TMS(1)	5260507	4202704	6055536	5499939	4429115	6843592	5140265	5875251	5254671.443	5572056.008	776360.8303	1033035.722
Lysine-4TMS	232805	218611	178507	245823	183406	235620	327136	292265	218936.5047	259606.9131	29153.82352	63268.25139
Glucose-meto-5TMS(2)	910859	743728	1022639	946935	731100	1202572	883462	1014008	906040.34	957785.4495	117807.9134	199993.0701
Tyrosine-3TMS	11307	9699	9988	13066	11367	10756	19039	17080	11015.23237	14560.19231	1535.984445	4126.189916
Sorbitol-6TMS	144679	125900	164786	124860	123565	192949	125474	89351	140056.3116	132834.8455	18834.83976	43376.75228
Xanthine-3TMS	51826	46441	39743	62282	49878	64733	52547	55840	50073.13964	55749.47074	9522.394151	6466.392185
Gluconic acid-6TMS	17625	12262	22208	21258	19863	25988	21216	20440	18338.32442	21876.64979	4506.408761	2796.223726
Palmitic acid-TMS	86529	140016	64488	61882	68838	64951	48715	58335	88228.69763	60210.0165	36252.17363	8804.679284
Uric acid-4TMS	11039	10666	12031	8803	21712	28697	17625	19875	10634.82289	21976.88778	1350.578239	4781.428404
Inositol-6TMS(2)	334931	417479	346839	359912	372970	562277	383622	410536	364790.537	432351.1557	36577.48475	88047.76216
Ribose 5-phosphate-meto-5TMS(1)	38944	28334	32419	26826	55696	82576	64725	74927	31630.90104	69481.02069	5417.678368	11744.42073
Stearic acid-TMS	53392	126340	47636	42212	46540	71659	41701	49358	67395.11441	52314.46509	39561.00676	13278.49684
Tryptophan-3TMS	115691	91992	91951	113046	102824	122334	154462	161533	103170.1568	135288.0752	12975.75702	27556.88016
Fructose 6-phosphate-meto-6TMS	307102	160955	420103	191870	226699	441525	265786	421286	270007.5775	338824.0217	118184.3689	108403.8819
Glucose 6-phosphate-meto-6TMS(1)	669686	359146	876425	428630	467099	892978	553108	863271	583471.7872	694113.88	236327.6663	215700.0151
Glucose 6-phosphate-meto-6TMS(2)	103067	53237	133937	61831	74000	138517	89280	147721	88017.97832	112379.385	37552.0257	36233.90526
Inosine-4TMS	266820	207200	295579	268746	346064	417187	354948	460582	259586.3188	394695.1676	37309.99837	54134.66095
Sedoheptulose 7-phosphate-meto-7TMS	50160	104126	52864	109824	55235	53261	52772	100239	79243.42467	65376.73844	32124.8112	23265.75112
Maltose-meto-8TMS(1)	65107	80172	84889	54705	19426	54085	41840	45552	71218.15106	40225.87277	13869.80992	14783.81083
Inosine monophosphate-5TMS	267357	144399	218416	87743	332235	322621	348172	316632	179478.9507	329915.0867	79340.4282	13763.70517
